# Corrosion Cast and 3D Reconstruction of the Murine Biliary Tree After Biliary Obstruction: Quantitative Assessment and Comparison With 2D Histology

**DOI:** 10.1016/j.jceh.2021.12.008

**Published:** 2021-12-20

**Authors:** Beate Richter, Sarah Zafarnia, Felix Gremse, Fabian Kießling, Hubert Scheuerlein, Utz Settmacher, Uta Dahmen

**Affiliations:** ∗ Department of General, Visceral and Vascular Surgery, Experimental Transplantation Surgery, University Hospital Jena, Drackendorfer Strasse 1, 07747, Jena, Germany; † Institute for Experimental Molecular Imaging, RWTH University Hospital Aachen, Templergraben 55, 52056, Aachen, Germany; ‡ Fraunhofer Institute for Digital Medicine MEVIS, Max-von-Laue-Str. 2, 28359 Bremen, Germany; § Clinic for General, Visceral and Pediatric Surgery, St. Vincenz Hospital Paderborn, Teaching Hospital of the University of Göttingen, Am Busdorf 2, 33098 Paderborn, Germany; ‖ Department of General, Visceral and Vascular Surgery, University Jena, Am Klinikum 1, 07747 Jena, Germany

**Keywords:** 3D reconstruction, biliary occlusion, biliary tree, corrosion cast, microfil, periportal segments, BD, bile duct, tBDT, bile duct ligation (using three sutures) with transection of the ligated extrahepatic bile duct between the middle and proximal sutures, BrdU, Bromodeoxyuridine, BT, extrahepatic and intrahepatic biliary tree, CC, Corrosion Cast using Batson No.17, CoH, Canals of Hering, DHC, Ductus hepatocholedochus, main extrahepatic bile duct, ehBD, extrahepatic bile duct, HE, Haematoxylin-Eosin, ihBD, intrahepatic bile duct, MV, Microfil®-MV, POD, postoperative day, 2D IHC, two-dimensional immunohistochemistry, 3D-reco, three-dimensional reconstruction, μCT, micro Computer Tomography (micro-CT)

## Abstract

**Background:**

Obstructive cholestasis can lead to significant alterations of the biliary tree depending on the extent and duration of the biliary occlusion. Current experimental studies reported about advanced techniques for corrosion cast and 3D reconstruction (3D-reco) visualizing delicate microvascular structures in animals. We compared these two different techniques for visualization and quantitative assessment of the obstructed murine biliary tree with classical 2D histology.

**Methods:**

Male mice (n = 36) were allocated to 3 different experiments. In experiments 1 and 2, we injected two different media (Microfil© for 3D-reco, MV; Batson’s No.17 for corrosion cast, CC) into the extrahepatic bile duct. In experiment 3 we sampled liver tissue for 2D histology (HE, BrdU). Time points of interest were days 1, 3, 5, 7, 14, and 28 after biliary occlusion. We used different types of software for quantification of the different samples: IMALYTICS Preclinical for 3D scans (MV); NDP.view2 for the digital photography of CC; HistoKat software for 2D histology.

**Results:**

We achieved samples in 75% of the animals suitable for evaluation (MV and CC, each with 9/12). Contrasting of terminal bile ducts (4th order of branches) was achieved with either technique. MV permitted a fast 3D-reco of the hierarchy of the biliary tree, including the 3rd and 4th order of branches in almost all samples (8/9 and 6/9). CC enabled focused evaluation of the hierarchy of the biliary tree, including the 4th to 5th order of branches in almost all samples (9/9 and 8/9). In addition, we detected dense meshes of the smallest bile ducts in almost all CC samples (8/9). MV and CC allowed a quantitative assessment of anatomical details of the 3rd and 4th order branches of almost every sample. The 2D histology identified different kinetics and areas of proliferation of hepatocytes and cholangiocytes. Complementary usage of 3D-reco, corrosion casting and 2D histology matched dense meshes of small bile ducts with areas of intensive proliferative activity of cholangiocytes as periportal proliferative areas of 4th and 5th order branches (∼terminal bile ducts and bile ductules) matched with its morphological information the matching assessment of areas with increased proliferative activity (BrdU) and a partial quantification of the characteristics of the 4th order branches of the biliary tree.

**Conclusion:**

The 3D-reco and corrosion casting of the murine biliary tree are feasible and provide a straightforward, robust, and reliable (and more economical) procedure for the visualization and quantitative assessment of architectural alterations, in comparative usage with the 2D histology.

Occlusive cholestasis is associated with significant alterations in the hepato-biliary architecture depending on the extent and duration of the biliary occlusion.^1^, ^2^, ^3^, ^4^, ^5^, ^6^, ^7^ For decades the classic 2D histology was accepted as the gold standard for morphological analysis.^1^^,^^2^^,^^4^, ^5^, ^6^, ^7^ Since the upgrade to 3D histology demands an enormous effort (e.g., hardware, software algorithm, personnel, time, funding), 3D histology is still a technique limited to highly specialized laboratories. Currently, confocal microscopy together with (spatial) 3D reconstruction (3D-reco) of small structures in mouse livers revealed details of biliary remodeling after biliary occlusion of unprecedented detail.^3^^,^^8^^,^^9^ In addition, the micro-CT-scan technique (μCT) evolved to a standard procedure in experimental medicine and an increasing number of studies showed excellent visualization of even fine structures in small animals like mouse brain or murine liver vasculature^8^^,^^9^^,^^10^^,^^11^^,^^12^.

Since most of these methods require an enormous effort, our aim was to establish a fast, straightforward, robust, and reliable (and more economical) procedure for the 3D-reco of the murine biliary tree during obstructive cholestasis. We used two different techniques: radiopaque Microfil®-MV (MV) for 3D-reco using micro-CT (μCT) and corrosion cast (CC) for focused imaging using a digital-stereo microscope. We evaluated these methods regarding visualization of the hierarchy of the murine biliary tree and quantitative assessment in comparison with classical 2D histology.

## Materials and Methods

### Experimental Design

We included three experiments in this study.

**Figure 1:**
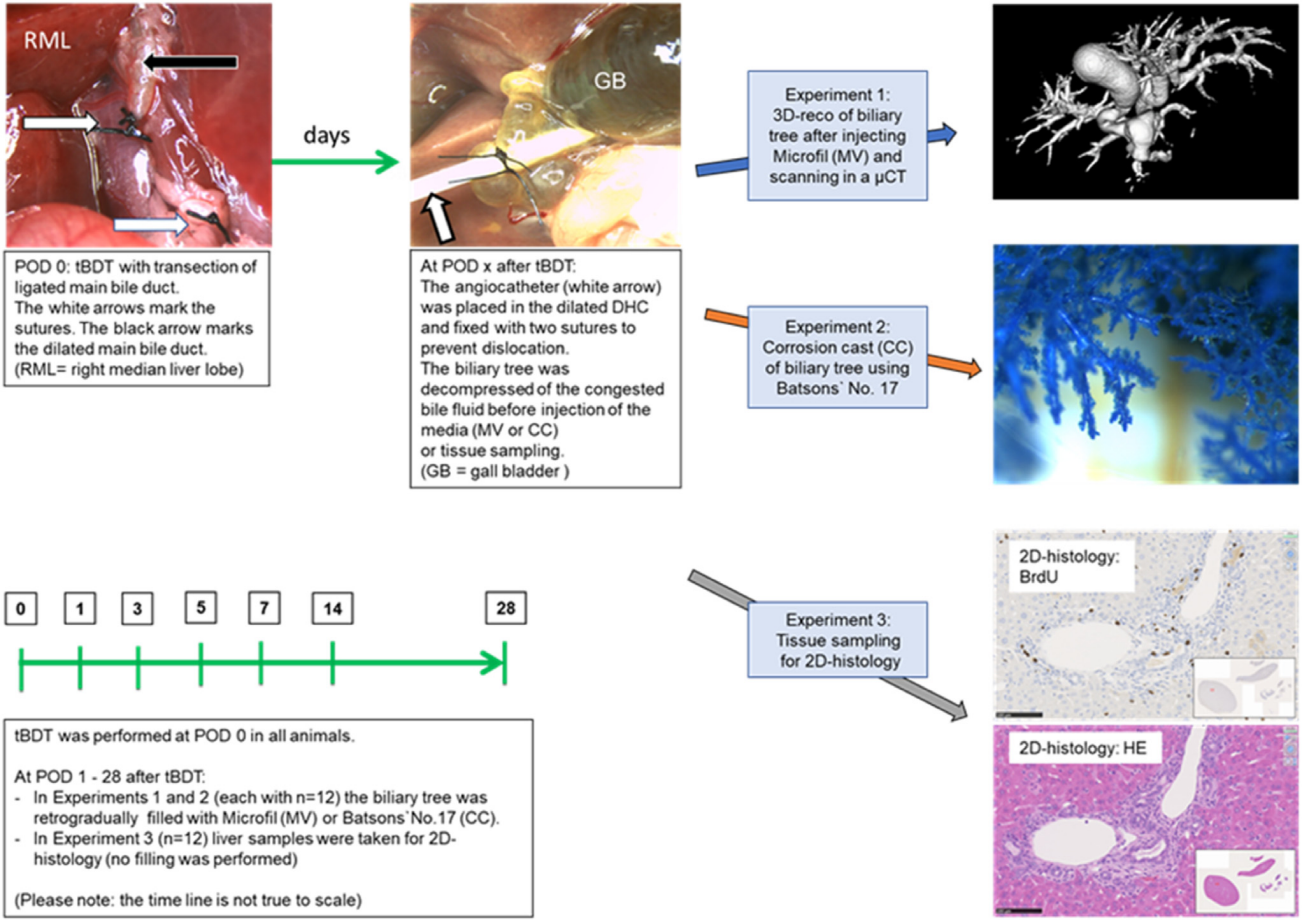
Experimental design comparing corrosion cast (CC) and 3D-reco (MV) of the murine biliary tree during occlusive cholestasis with 2D histology.

All animals (n = 36) received in the first operation a ligation and transection of the ligated extrahepatic main bile duct (tBDT) to induce occlusive cholestasis. At six time points after tBDT the animals were randomly assigned to one of three different experiments (see Figure 1). Time points of interest were postoperative days (POD): 1, 3, 5, 7, 14, 28.

**Experiment 1** (n = 12) was performed to obtain whole liver samples for 3D-reco after the biliary tree was retrogradely filled with the contrast medium Microfil (MV).

We included HE staining of selected samples of all lobes as an “internal quality control” to confirm filling of the biliary tree with MV and to identify potential parenchymal extravasates. In addition, we compared the extent of the histologically detected bile ducts with those visualized by the 3D-reco of MV samples. Therefore, samples of all liver lobes were taken after completion of the 3D reconstruction of the μCT-Scan. After paraffin embedding, 4 μm sections were subjected to Hematoxylin-eosin staining and used for routine histological examination.

**Experiment 2** (n = 12) was dedicated to obtain corrosion casts of the intrahepatic biliary tree by using the respective kit (Batson’s No.17) (CC). The solid corrosion casts prevented a transfer into processible samples for histology. Consequently, we could not perform additional histological analyses of the CC samples.

**Experiment 3** (n = 12) was designed to obtain liver tissue samples after tBDT for histological and immunohistochemical evaluation of the mouse liver. These samples were used as controls. Thus, no additional injection of any mixture was performed in these animals. Samples were stained with Hematoxylin-eosin (HE) and Bromodeoxyuridine (BrdU) to compare the morphological alterations with the results of the other two experiments.

### Animals

All surgical procedures were performed in inbred male mice (C57BL/6N, Charles River, Germany) aged 9–10 weeks (body weight 25–28 g). Mice were fed a standard laboratory diet with water and mouse chow ad libitum until harvest. All animals were kept under constant environmental conditions with a 12 h light–dark cycle in a conventional animal facility using environmentally enriched type IV cages in groups of 2–3 mice. All procedures and housing of the animals were carried out according to the German Animal Welfare Legislation and approved by the local authorities (Landesamt für Verbraucherschutz Thüringen).

### Surgical Technique

All interventions were performed at day-time under inhalation of 1.5–2.0% isoflurane mixed with pure oxygen at a flow of 0.3 L/min (isoflurane vaporizer, Sigma Delta, UK) in a dedicated S1 operation room. All procedures were done under an operating microscope (Leica, magnification 10–25×, Germany) to ensure the preservation of the branches of the hepatic artery and portal vein. We induced total biliary occlusion in 36 male mice (C57BL/6N, 25–28 g) as described before.^13^ We performed a **t**otal **b**ile **d**uct ligation and **t**ransection of the ligated main bile duct between the two distal ligatures (**tBDT**). We used three ligatures for ligation of the main bile duct and transected the ligated bile duct between the two distal ligatures. The transection of the ligated main bile duct prevents the formation of biliary collaterals or recanalization due to loosening of ligatures, compared to bile duct ligation using single or double ligation of the main bile duct without transection (BDL).^14^, ^15^, ^16^, ^17^, ^18^We used 12 mice for each experiment. No mice died before the intended sacrifice date. Detailed description of the surgical procedure and postoperative observation and analgetic treatment of the animals is given in the supplement.

### Specimen Preparation

At the indicated time points of interest, the animals were randomly assigned to one of the three experiments and anesthetized as described above. The abdomen was reopened, and the animals were sacrificed via exsanguination (Vena cava inferior). Subsequently, we cannulated the dilated extrahepatic bile duct with a 24-gauge catheter for decompression of the biliary fluid and injection of the media. The catheter was fixed with two sutures (6–0 silk), preventing dislocation.

For experiments 1 and 2, the mixtures of MV or CC were retrogradually injected into the biliary tree using a manually pressure controlled method as described previously in more detail.^10^^,^^12^ The quality of the injection was monitored by visual control (naked eye and Leica microscope) throughout the procedure. The injection was stopped when the colored mixture appeared on the surface of the liver lobes. Depending on the body and liver weight and the duration of biliary obstruction, a mean volume of 2–2.5 ml of MV or CC was needed. We did never inject microfil and corrosion cast in the same animal. We refrained from the inclusion of another casting procedure with the conversion of the “closed” biliary system into an “open” system (e.g., by cutting the liver surface; by dividing the liver lobes) to avoid any unintended “artificial variations” of the biliary anatomy. Furthermore, we refrained from the usage of SEM (especially of CC samples) since one of our objectives was to establish a straightforward concept to obtain a 3D visualization of the murine biliary tree.

All MV samples were placed in 4% formalin for fixation until scanning. All CC samples were used for focused imaging (Leica microscope and Canon).

For experiment 3, a sample from each liver lobe was taken for formalin fixation, embedding, and staining. In these animals, only tBDT but no injection of any mixture into the biliary tree was performed.

### 3D Reconstruction of the Murine Biliary Tree After tBDT Using μCT

Microfil®-MV is a commercial silicone-based injection compound (FlowTech Inc., USA) and cures to a soft radiopaque 3D cast within 3–4h after application. According to the manufacture’s description and after preliminary bench-site experiments in our laboratory, we used 2 ml of colored component (MV120, blue) + 2 ml of diluent + 200 μl of curing agent to obtain an MV-mixture reliably curing within ∼60min after mixture. The organs filled with MV were left in-situ for another 12 h at 4 °C (refrigerator) until polymerization was completed to preserve organ shape. The explanted whole liver sample was fixed in formalin until scanning. Samples were scanned in a μCT enabling a digital 3D-reco of the contrasted biliary tree using the IMALYTICS Preclinical software.^19^^,^^20^

### Corrosion Cast of the Murine Biliary Tree After tBDT

Batson`s No.17 (Corrosion Cast, Polysciences, USA) consists of a multicomponent polymer (methyl methacrylate) with an added colored pigment (green, blue, or red) and cures to a durable and solid cast within 4–6 h after injection. Whole livers filled with Batson’s No.17 (CC) were immediately cooled in rinsing cold water for at least 2 h preserving the organ from damages of the exothermic curing process of CC. Afterward, the samples were kept for another 12 h at 4 °C (refrigerator) until polymerization was completed to preserve organ shape. The remaining soft tissue was removed using the maceration procedure as described in the manufacturer’s instructions. The maceration process included two washing steps to remove excess soft and fibrous tissue and was finished within 3 days. After maceration, the specimens dried within 24 h. Finally, the specimens were used for focused imaging using a digital stereomicroscope (Leica M60 + IC80HD) and macro-pictures (Canon EOS 450D, EFS 18–55 mm). During imaging, the samples were placed on a millimeter paper for calibration enabling comparative examinations.

### *Ex vivo* Micro-Computed Tomography Scan (μCT-Scan)

The specimens fixed in formalin were scanned by μCT (Tomoscope Duo CT, CT Imaging GmbH, Erlangen, Germany) using the scan-protocol HQD-6565-390-90 (voltage of 65 kV, current of 0.5 mA) with 720 projections (approx. 1032 × 1012 pixels) during one full rotation (360°) with a scanning time of 90s per subscan.^21^^,^^19^^,^^20^ In all samples, we used the gall bladder as a landmark to assure identical orientation of the 3D-reco.

### Histology and Immunohistochemistry

#### Hematoxylin-Eosin Staining (HE) and Bromodeoxyuridine Staining (BrdU)

We used HE for histologic and morphological analysis (Experiments 1 and 3) of the liver tissue and BrdU for the detection of the proliferation indices of hepatocytes and cholangiocytes in the same section (Experiment 3). We always took samples from the middle part of every liver lobe perpendicular to the main vessels assuring evaluation of comparable areas of the liver lobes in all animals. Sections of 4 μm thickness were cut after paraffin embedding. After staining, all slides were digitalized using a slide scanner (Nanozoomer, Hamamatsu Electronic Press Co., Ltd, Iwata, Japan).

For morphological assessment, we used the nomenclature of the biliary tree and the ductular reaction due to biliary occlusion according to Li and Roskams.^1^^,^^2^

#### Quantification of Proliferation

Proliferative activity of hepatocytes and cholangiocytes per bile duct was determined using the Histokat software developed at Fraunhofer Mevis (Dr. Homeyer, Fraunhofer MEVIS, Bremen, Germany). This software can be trained to either include or exclude elements in an image and can be used for batch analysis of images with similar staining properties. The software was kindly provided by Fraunhofer-Institute (Fraunhofer MEVIS, Bremen, Germany).^22^

Detailed descriptions of the staining methods are listed in supplement.

### Quantification of Visualized Segments of the Murine Biliary Tree

The IMALYTICS Preclinical software permitted the segmental determination of diameter, length, and branching frequency of the digital 3D scans of the MV samples.

The digital photography of CC samples (Canon and Leica microscope) were analyzed (segmental diameter and length) using the software NDP.view2 (Hamamatsu Electronic Press Co., Iwata, Japan).

The digitalized 2D-histology slides were analyzed (morphometry, segmental diameter) with the software NDP.view2 (Hamamatsu Electronic Press Co., Iwata, Japan).

### Statistical Analysis

The data are expressed as mean ± standard deviation (SD) if not indicated otherwise.

## Results

### Contrasting the Biliary Tree was Successful in 75% of the Animals (9/12 Subjected to MV or CC, Respectively) Using the Manually Controlled Retrograde Injection Technique

In three animals from both groups (MV and CC), we faced severe problems with an enormously increased filling pressure in three samples per group (MV, CC: n = 1 at POD 1, 3, and 5, respectively), possibly due to incomplete decompression of the bile fluid or micro air bubbles in the injection solution (Table 1, 2).

**Table 1:** Qualitative Assessment of Either Method Concerning Visualization of the Murine Biliary Tree in Obstructive Cholestasis.

	MV (3D scan)	CC	2D histology
Method already established in literature?	not for biliary tree in mice	not for biliary tree in mice	Yes
Problems with mixtures/methods	high viscosity of mixture; micro air bubbles in mixture with risk of incomplete filling		
Problems during injection	possible due to incomplete biliary decompression prior to injection: - changing resistance during manually controlled retrograde filling of biliary tree, - irregularly increased filling pressure (until day 28 after tBDT)		not applicable since no injection was performed in this group
Sample outcome (n = 12 animals per group)	9 out of 12, 75%	9 out of 12, 75%	12 out of 12, 100%
Complete hierarchy of biliary tree visible	in 7/9, ∼78%	in 7/9, ∼78%	no
Smallest identifiable biliary segment	in 9/9, 100%: 3rd order branches (∼segmental bile ducts), in 7/9, ∼78%: 4th order branches (∼terminal bile duct)	9/9, 100%: 4th order branches (∼terminal bile duct)	12/12, 100%: 4th order branches (∼terminal bile duct)
Quantification software	IMALYTICS preclinical (developed by Dr. Gremse and Prof. Kiessling, Molecular Imaging, RWTH Aachen)	NDP.view2 (free software, Hamamatsu Electronic Press Co., Japan)	HistoKat (developed by Dr. Homeyer, Fraunhofer MEVIS, Germany)
Limitations	truncated segments, possible due to incomplete filling	fragile samples digitalized by focussed imaging,	2D limitation, restricted evaluation area (e.g., slide area)
Benefits	3D data sets, fast overview of biliary hierarchy	richness in detail, focussed imaging enabled reliable identification of even small biliary segments.	standardized staining protocols, digitalized slides/data sets via histo-scanning

tBDT, bile duct ligation; CC, Corrosion Cast; MV, Microfil®-MV.

**Table 2:** Assessment of Either Method Concerning Benefits for Visualization of the Murine Biliary Tree in Obstructive Cholestasis.

	MV (3D scan)	CC	2D histology
Examples for beneficial complimentary use of either method: - MV and CC	Similar segmental spike and twisted-like alterations of segmental and terminal bile duct walls at similar time points (day 3, 5, 7)		
- CC and 2D histology		Dense meshes of small bile ducts (CC) and the periportal biliary proliferates (2D histology) became the dominant findings at similar time points (day 14 and 28)	
Differences between methods:	Evaluation of changes in proportional relations: - only of single biliary segments: 2D histology - of multiple biliary segments: MV and CC - of almost all biliary segments: CC Determination of branching frequency: predominantly in CC, limited in MV samples due to incomplete filling Assessment of morphological hepato-biliary alterations in 2D histology (e.g., with relation to a specific molecular target: proliferative activity using BrdU) Assessment of biliary architectural alterations predominantly in MV and CC		
Recommendation	Fast visualization of the shape of the biliary tree. Determination of structural alterations on the macro-scale, mostly, including 4th order branches.	Focussed imaging detected richness of detail even of small segments of the biliary tree on the meso-scale. Timely determination of biliary alterations, including 4th order branches.	Morphological examination (HE) on the microscale. Relates morphological structure to molecular events by immunohistochemical visualization of the molecular target (e.g. proliferative activity by BrdU)

BrdU, Bromodeoxyuridine; CC, Corrosion Cast; HE, Haematoxylin-Eosin; MV, Microfil®-MV.

In these three MV samples, the 3D-reco showed incomplete filling of the biliary tree and substantiated the suspicion that micro air bubbles prevented complete filling of the biliary tree (see Figure 1 in supplement). The HE staining of the residual nine MV samples (see Figure 2 in Supplement) revealed that MV remained inside the bile ducts and was not located in any vessel (not inside portal veins or liver veins or hepatic arteries). In two samples of the residual nine samples (1 × POD 3, 1 × POD 14), we identified particles of MV in the hepatic parenchymal tissue as a sign of extravasation (2/9, ∼22%).

In the three “high-pressure” CC samples, we found truncated segments of lobar and segmental bile ducts indicative of incomplete filling, potentially due to incomplete biliary decompression or micro air bubbles.

### 3D-Reco (MV) and Corrosion Casts (CC) Visualized Different Details of the Biliary Tree

**Figure 2:**
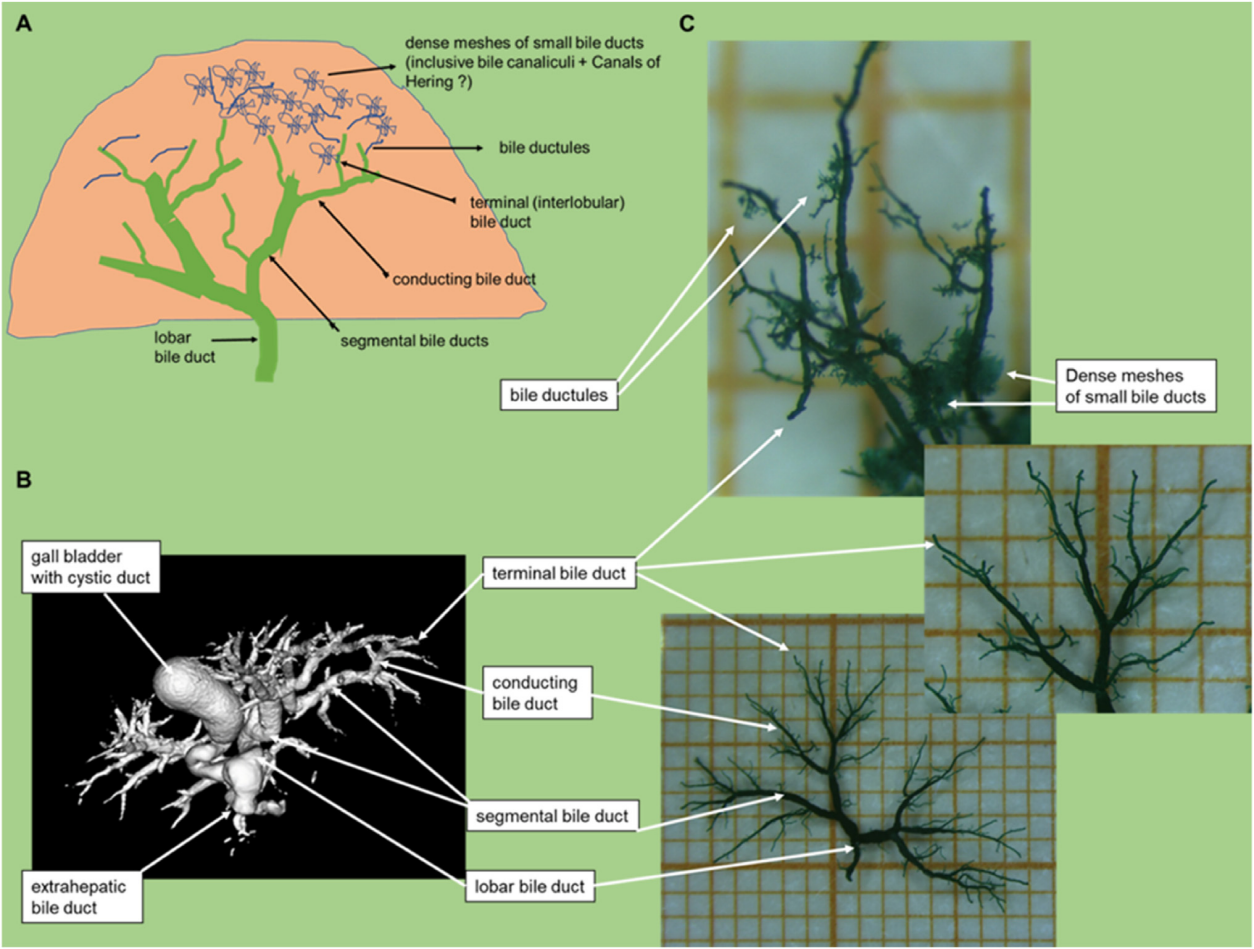
A-C: Comparison of contrasted segments of the murine biliary tree using MV for 3D-reco and Batson’s No.17 for corrosion cast (CC).

**Figure 3:**
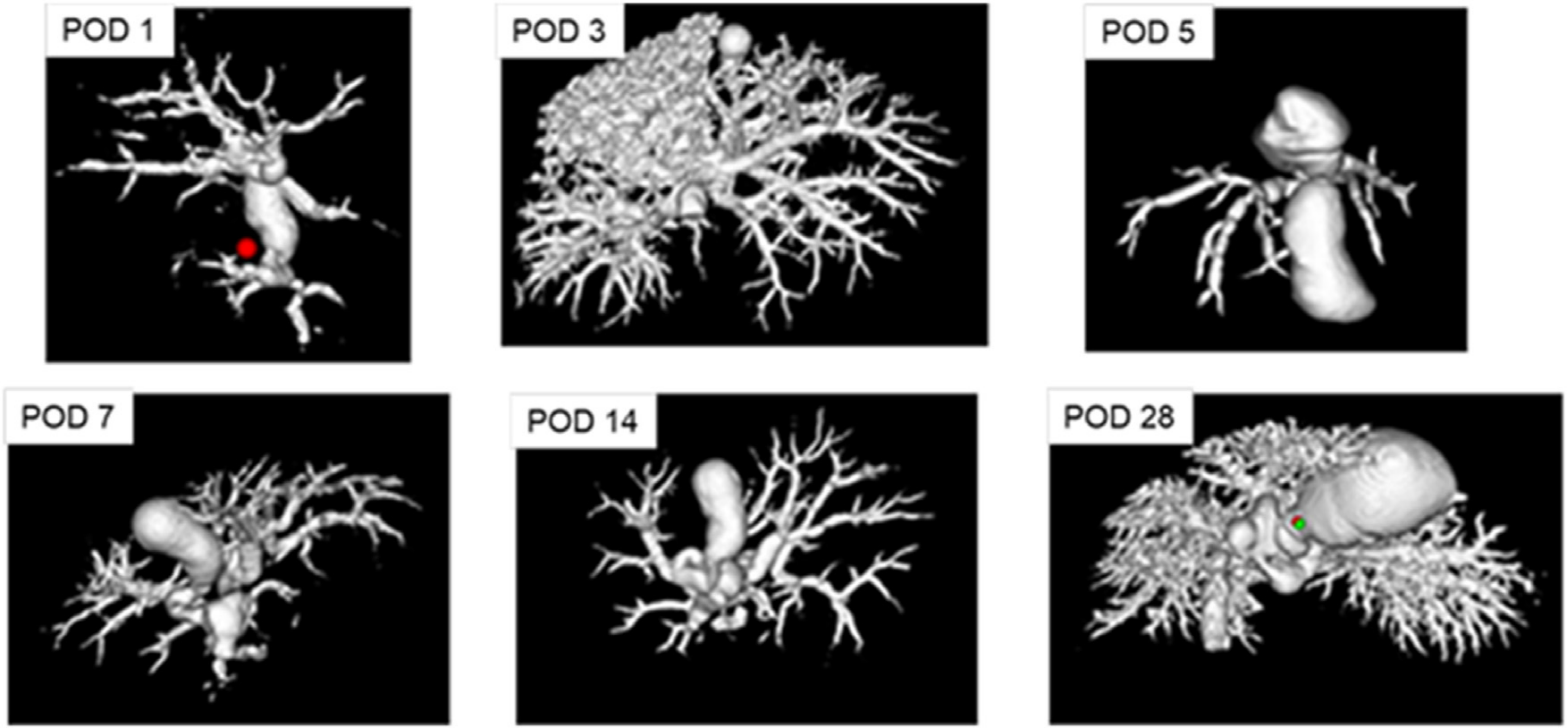
3D reconstruction (MV) of the biliary tree, including the terminal bile ducts, was feasible in the majority of the samples.

**Figure 4:**
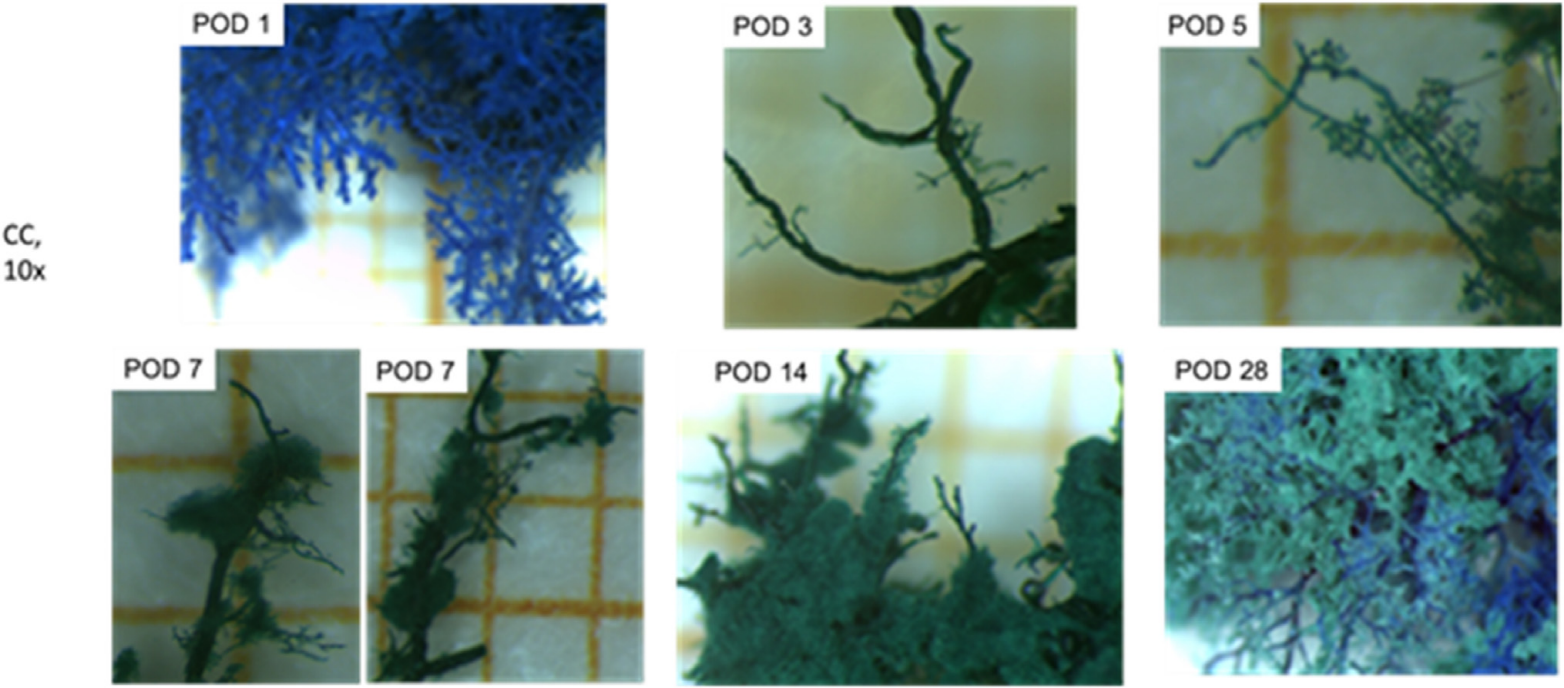
Images of corrosion casts of murine liver samples (tBDT) at six different time points after biliary obstruction.

MV and CC showed the hierarchy of the biliary tree even down to the 4th order branches (∼terminal bile ducts) at almost all time points. In all remaining nine samples of MV, the reconstruction of the complete intrahepatic biliary tree, including branches of 3rd order (segmental bile ducts; acc. to^1^^,^^2^), was possible (see Figure 3, Figure 4; Table 1, Table 2, Table 3). In the majority of the samples, we identified even branches of 4th order (terminal bile ducts) (6/9, ∼67%). In all remaining CC samples (n = 9) we detected the terminal bile ducts (see Figure 2, Figure 4, Figure 6), and in most samples, even dense meshes of terminal bile ducts with smaller bile ducts (8/9, ∼89%). Furthermore, MV and CC visualized twisted-like and spike-like wall alterations of different extents in dilated bile ducts of different orders (e.g., segmental and terminal bile ducts) at similar time points (day 3, 5, 7 after tBDT). In addition, corrosion cast allowed focused assessment of smaller bile ducts (e.g., segmental bile duct vs. terminal bile duct) (see Figure 2, Figure 4) and showed repeatedly dense meshes of small bile ducts.

**Table 3:** Results of Quantitative Assessment for Visualization of the Murine Biliary Tree After tBDT (n = 2 per Time Point and Method).

	POD 1	POD 3	POD 5	POD 7	POD 14	POD 28
	mean ± stdev.	mean ± stdev.	mean ± stdev.	mean ± stdev.	mean ± stdev.	mean ± stdev.
**3D-reco (MV)***of 4th order branches (terminal bile duct)*						
number of visible branches	0.00 ± 0.00	20.43 ± 3.99	8.00 ± 2.55	20.00 ± 2.16	18.33 ± 2.81	22.67 ± 2.87
length [μm]	0.00 ± 0.00	5.67 ± 1.49	9.67 ± 0.47	14.83 ± 2.48	16.83 ± 2.67	12.67 ± 1.89
diameter [μm]	0.00 ± 0.00	44.33 ± 6.20	39.86 ± 7.94	60.33 ± 6.47	63.17 ± 6.47	67.67 ± 10.92
**Corrosion cast (CC)***of 4th order branches (terminal bile duct)*						
number of visible branches	29.89 ± 2.92	24.60 ± 2.94	21.43 ± 1.68	23.17 ± 3.34	26.17 ± 3.18	33.33 ± 6.97
length [μm]	8.55 ± 1.37	10.22 ± 2.82	13.43 ± 3.42	16.17 ± 2.27	25.50 ± 3.86	23.50 ± 3.59
diameter [μm]	21.27 ± 7.19	45.56 ± 4.97	39.57 ± 3.89	56.00 ± 3.42	57.50 ± 4.79	60.00 ± 4.08
**2D histology***of 4th order branches (terminal bile duct)*						
Number of bile ducts per portal field	7.75 ± 2.01	7.14 ± 1.75	8.83 ± 1.34	15.50 ± 3.99	18.33 ± 8.33	18.67 ± 6.87
Diameter of bile ducts per portal field [μm]	14.36 ± 2.30	39.53 ± 1.63	31.94 ± 1.12	55.28 ± 1.87	51.57 ± 1.56	131.69 ± 39.67

CC, Corrosion Cast; MV, Microfil®-MV; 3D-reco, three-dimensional reconstruction.

**Figure 5:**
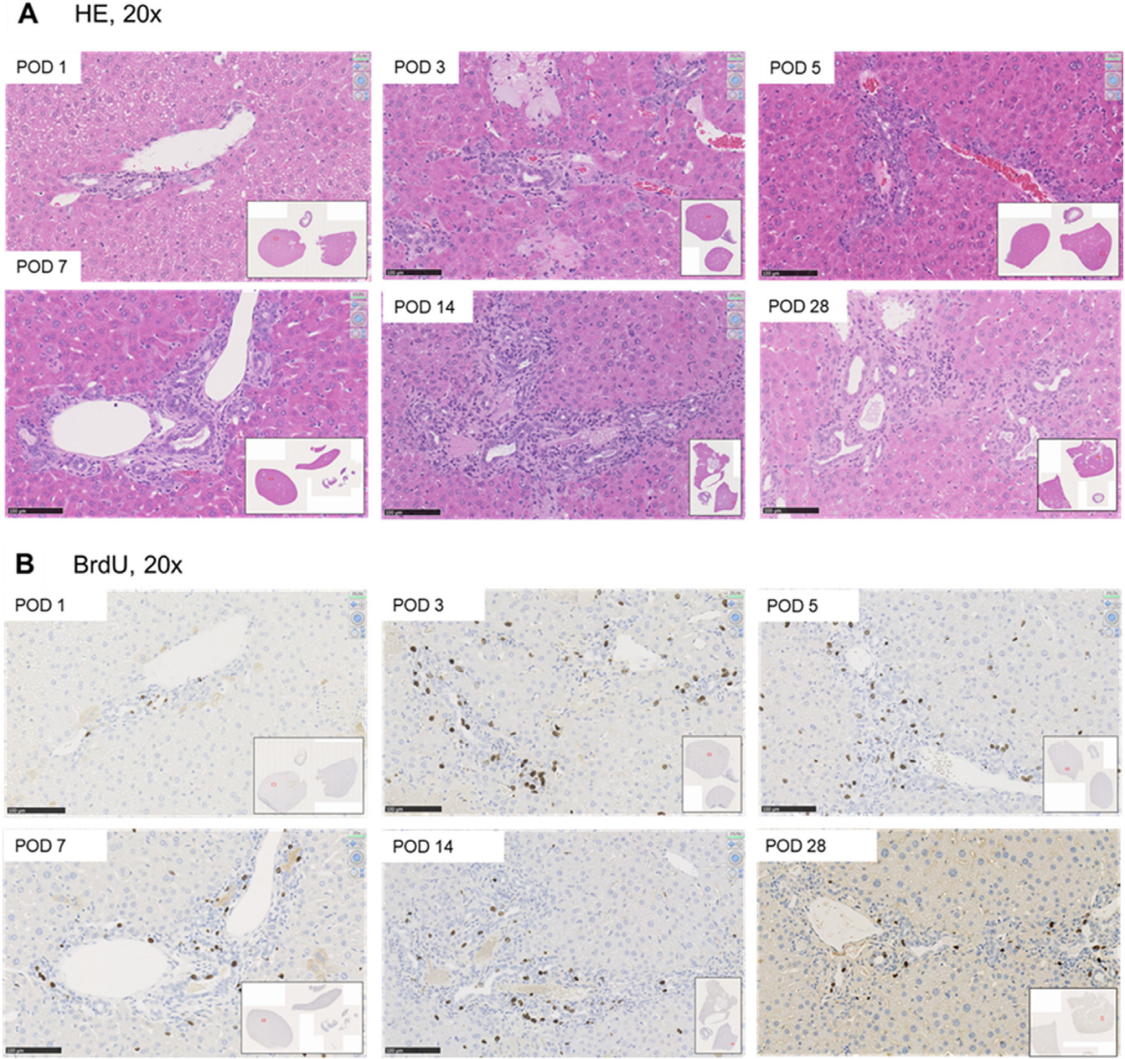
A–B: 2D histology of the murine liver after biliary occlusion.

**Figure 6:**
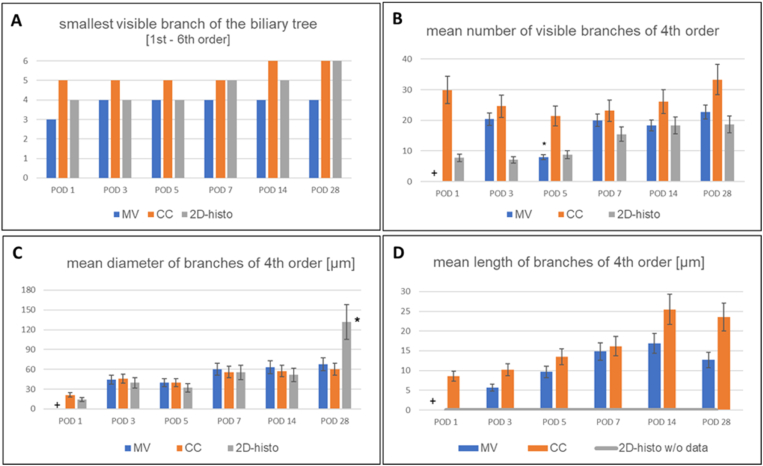
A-D: Comparison of the methods` quantification results focusing on branches of 4th order of the murine biliary tree after bile duct ligation.

The 2D histology visualized the enlargement of the portal fields indicative of the ductular reaction. We noticed a continuous increase in the number of bile ducts per extended portal field and extraportal biliary proliferates. The distinct increase in diameter of portal bile ducts was related to an increased number of longitudinally sectioned bile ducts at POD 28. We detected increased proliferative activity of cholangiocytes (BrdU) in the portal and extraportal biliary proliferates with peaks at POD 3 and 14 (see Figure 5, Figure 3 in supplement; and Table 3). Whereas the proliferative activity of the hepatocytes constantly increased until day 7, followed by fast reduction to nearly normal values at day 28 (∼end of observation) (see Figure 3E+F and Table 1 in supplement). In consequence of the enlargement of the “biliary compartment” due to tBDT, we noticed a stepwise reduction of the hepatocellular compartment down to 85% (relative area) until day 28 (see Figure 3E and Table 1 in supplement). Using 2D histology, we could not differentiate between elongation and kinking of the bile ducts in contrast to the formation of a network-like structure due to enhanced branching as the underlying reason.

### 3D-Reco and Corrosion Casts Allowed Segmental Quantification of the Biliary Tree to a Different Extent

**Figure 7:**
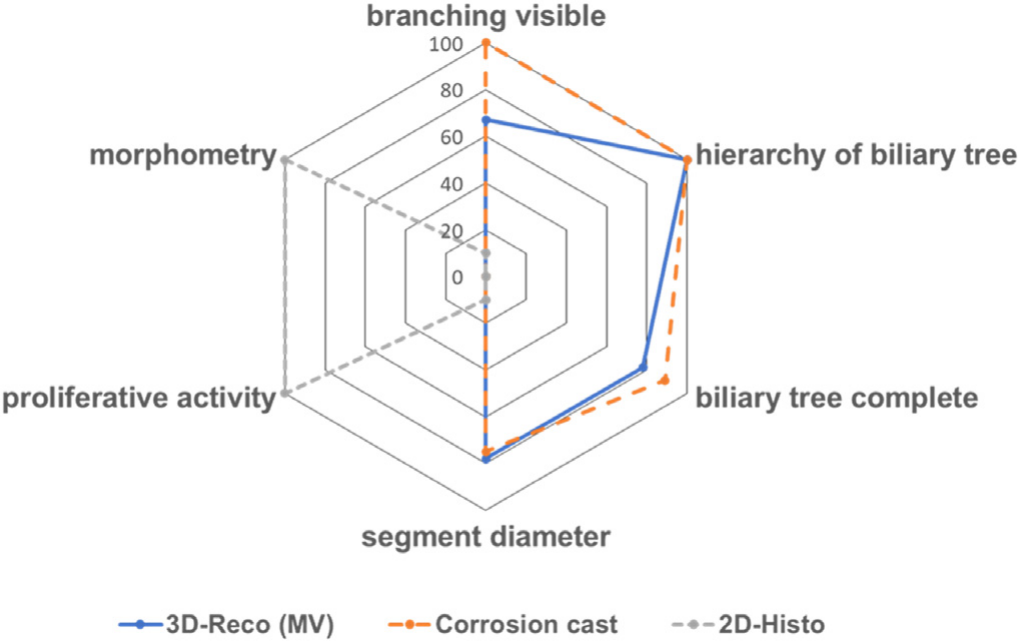
Spider net with a comparison of the potentials (strengths and limitations) of MV, CC, and 2D histology.

The three methods provided the visualization of branches down to the 4th order (terminal bile ducts; according to the nomenclature of^1^^,^^2^) (see Figure 6, Figure 7; and Table 1 in supplement; Table 1, Table 2, Table 3). Therefore, we focused on this segment of the biliary tree for comparing the strength and limitations of the methods regarding (quantitative) morphometry (hierarchy of the biliary tree, diameter, length, visible number of branches, morphometric analysis). We refrained from volumetric measurements due to the irregular structure of the bile ducts.

The quantification of the MV samples was mostly limited by truncated branches of 4th order at POD 1 and of even smaller segments at all time points. CC allowed a nearly complete quantification (hierarchy, diameter, length, visible branches) of the branches of 4th order (terminal bile ducts). Smaller biliary segments were identified as branches of 5th order (∼bile ductules, see Figure 2A–B) or dense meshes of small bile ducts (∼possibly including bile canaliculi and Canals of Hering, see Figure 2A–B, Table 1, Table 2, Table 3) (acc. to the nomenclature of^1^^,^^2^).

As expected, the 2D histology revealed its strength in morphologic analysis (see Figures 6 and 7; and Table 3). The 2D histology allowed identification of branches from 4th order, partly down to 6th order at the later time points after tBDT (day 14, 28) (see Figure 6A-C). The restriction to 2D prevented the determination of the length of branches (see Figure 5, Figure 6D).

## Discussion

Visualization of the obstructed murine bile duct is important for better understanding the pathophysiology and dynamics of biliary obstruction. Since it is rarely examined, we designed this comparative study.

In preliminary tests, we faced severe problems with the filling pressure to achieve homogenous filling of the biliary tree, although we followed the few available recommendations regarding the intrabiliary pressure after biliary occlusion.^23^^,^^24^ Finally, we obtained reliable and high-quality samples only by using the manually controlled filling technique. A few authors described this technique for preparing casts of the hepatic vasculature and biliary tree in mice before.^10^^,^^11^^,^^12^ Interestingly, other authors described pump-based filling techniques as successful even for the murine hepatic vasculature.^25^^,^^26^ Since the biliary tree represents a “closed system” with a fluid of high viscosity and low velocity, it might be possible that the injection techniques for the biliary tree require special customization of the setup. Furthermore, we noticed a changing resistance during filling that was easy to adapt to with the manually controlled pressure. This was confirmed by exceptionally low numbers of ruptured and truncated segments of the biliary tree in our experiments. Therefore, we believe that the hand pressure technique is the appropriate filling method for the murine biliary tree if very viscous media are used. Facing these results, these advantages are overcoming the disadvantage of a nonstandardizable technique (manual filling).

In our study, CC was best suited for visualization of the hierarchy of the whole biliary tree. We repeatedly achieved the filling of the tree, including the finest structures like dense meshes of small bile ducts. This impressive richness in detail of the visualization enabled a quantitative assessment, including smaller bile ducts (e.g., terminal bile ducts and bile ductules). However, one limitation is the extreme fragility of the resulting casts requiring extremely gentle handling. The MV contrasting was best suited to visualize the shape of the biliary tree with a fast determination of structural alterations on the macro-scale, mostly including branches of 4th order. The 2D histology was best suited – as expected – for the morphological examination (HE) on the microscale. It allowed to relate morphological structure to molecular events by immunohistochemical visualization of the molecular target, in our case BrdU as an indicator of the proliferative activity.

As mentioned before, first visualizations of small anatomical structures using MV or CC were demonstrated by several studies for the vasculature of solid organs in rodents (e.g., brain, heart, lung, liver, mesenteric vessels). Only a few of the experimental articles described details of the biliary tree, including small bile ducts using similar techniques in rat livers.^9^^,^^10^^,^^11^^,^^12^ Even smaller is the number of experimental articles addressing the visualization and quantification of biliary structures in obstructive cholestasis using MV or CC in mice.^2^^,^^8^^,^^14^ These few articles presented in part comparable results for MV or CC (e.g., visible hierarchy, number of branches, the diameter of segments) but only at one single time point after bile duct ligation. We obtained samples (MV + CC), allowing visualization and assessment of almost the complete murine biliary tree, including the smallest bile ducts at six different time points after bile duct ligation.

At present, only studies employing confocal microscopy for 3D reconstruction showed an exceptional richness of detail of small murine biliary segments.^2^^,^^3^^,^^8^^,^^9^ Most of the authors focused on the assessment of the periportal bile ducts (equal to branches of 4th order and terminal bile ducts/interlobular bile duct, acc. to^1^^,^^2^), since this biliary segment is known for its impressive proliferative activity and potential for adaptive remodeling. The biliary remodeling was mostly characterized by an increased branching frequency and rejoining of mostly new built branches into dense and complex meshes.^1^^,^^3^, ^4^, ^5^ The authors supposed that this “adaptive remodeling” and the consequently increased biliary surface serves different purposes: reduction of the intrabiliary segmental pressure by increasing the filling volume via elongation, and/or branching leading to network formation (e.g., biliary occlusion); or bypassing dysfunctional or remodeling biliary areas (e.g., toxins, ischemia, obstruction). Interestingly, these biliary remodeling mechanisms were described predominantly in mice.^3^^,^^8^^,^^9^^,^^14^ Our samples visualized similar characteristics of “adaptive remodeling” of the biliary tree. Dense meshes of small bile ducts (CC) and the extraportal biliary proliferates (2D histology) became the dominant findings observed at similar time points (POD 14 and 28) in most of the samples. Therefore, we believe that we visualized a similar phenomenon with either technique: dense meshes of newly built branches of terminal bile ducts and bile ductules. In another complementary usage of MV and CC, we observed similar segmental spike and twisted-like alterations of walls of segmental and terminal bile ducts at similar time points. Comparable bile duct wall alterations for mainly terminal bile ducts were recently described by confocal microscopy.^3^^,^^9^^,^^27^

Consequently, our study illustrates the complementary benefits of these three methods, especially for visualization and detection of intrahepatic architectural alterations of the small biliary segments after tBDT. We experienced MV and CC as fast, reliable, and economical techniques for 3D visualization since these methods do not require an intensive input of rare resources (e.g., animal, human, time, hardware, funding) after establishment. In connection with these favorable conditions, CC and MV could be used as “first-line diagnostic tools” to visualize other alterations using a fast and direct approach. In the next step, molecular details of the structural alterations could be determined by other methods, such as confocal microscopy. Such a step-up scenario preserves resources and may add new insights into the visualization of small structures in rodents (especially mice).

As the 3D visualization of the murine biliary tree is a rather novel research field in cholestatic liver diseases, more investigations are needed to refine the methods of 3D imaging in experimental settings.

The 3D-reco and corrosion casting of the murine biliary tree are feasible and contribute new aspects to the visualization of the architectural alterations of the biliary tree after bile duct ligation. Complementary usage of 3D-reco, corrosion casting, and 2D histology matched dense meshes of small bile ducts with areas of intensive proliferative activity of cholangiocytes as periportal proliferative areas of 4th and 5th order branches (∼terminal bile ducts and bile ductules).

## Credit authorship contribution statement

BR and UD designed the study, BR performed the study, made the 3D-reco of the μCT-Scans, analyzed the data and wrote the manuscript; SZ did the μCT-scan; FK, FG kindly provided their μCT for all scans and the software for 3D reconstruction of the μCT-Scans; SZ, HS, FK, FG, UD, US revised the manuscript; UD and US financed the study and publication.

## Conflicts of interest

The authors have none to declare.

## Acknowledgements

The authors would like to express their gratefulness for the excellent support of the technical co-workers: Ms. I. Jank for the organization of all the office work, Mrs. St. Lange for her animal care, Ms. B. Lanick for her elaborated bench-side experiments, Mrs. E. Oswald and St. Lange for the preparation of all the histological slides, and Ms. K. Schulze for staining and scanning of all the slides.

## Funding

This study was supported by the clinical research supporting program of the 10.13039/100012957University of Jena to B.R (“IZKF-Rotations program”, URL: http://www.izkf.uniklinikum-jena.de; committee with varying members of the University of Jena); and was funded by the 10.13039/501100002347German Federal Ministry of Education and Research (BMBF: http://www.bmbf.de) via the Virtual Liver Network, grant numbers 0315743 (SZ, FG, FK) and (0315765 (UD), as well as the DFG-funded Collaborative Research Centers CRC1382 (project ID 403224013 - SFB 1382, project Q1 (FG, FK). The funders had no role in study design, data collection and analysis, decision to publish, or preparation of the manuscript.
