# Supplementary material for: Corrosion Cast and 3D Reconstruction of the Murine Biliary Tree After Biliary Obstruction: Quantitative Assessment and Comparison With 2D Histology
Source: J Clin Exp Hepatol. 2021 Dec 20;12(3):755–66. doi: 10.1016/j.jceh.2021.12.008 (PMC9168744; doi:10.1016/j.jceh.2021.12.008)
Supplement: Multimedia component 1 [file mmc1.docx]

**Supplement:**

**List of tables and figures listed in supplement**

**List of Figures:**

**Figure 2:** 3D-recos of samples with incomplete filling of the murine biliary tree after tBDT.

**Figure 5:** “Internal quality control” using HE-staining of MV-Samples to identify MV inside bile ducts and to identify extravasates.

**Figure 8 A-F: Results of morphometric analysis of 2D-histology** using HE and BrdU. After tBDT the relative area of bile ducts per PF increased (**A**), due to slowly increasing diameter (**B**) and primarily to strongly increasing number (**C**)of bile per portal field. The cholangiocytes showed a proliferative activity (**D**) with two peaks at day 3 and day 7 after tBDT. The hepatocellular compartment was stepwise reduced (**E**) due to the biliary enlargement following biliary occlusion. The hepatocytes showed a constantly increasing proliferative activity (**F**) until day 28 (~end of observation) after tBDT.

**List of Tables:**

**Table 1:** Results of Morphometry and Immunohistochemistry of mice liver at different time points after tBDT (n=2 per time point). (in an extra file)

**Materials and Methods:**

**Surgical technique**

All interventions were performed at day-time under inhalation of 1.5-2% isoﬂurane mixed with pure oxygen at a flow of 0.3L/min (isoflurane vaporizer, Sigma Delta, UK) in a dedicated S1 operation room. At the end of the day the instruments were cleaned and sterilized in a commercial autoclave (Systec DE-23, Germany). All procedures were done under an operating microscope (Leica, magnification 10-25x, Germany) to ensure preservation of the branches of the hepatic artery and portal vein. All animals were weighed and then anaesthetized with 2% isoflurane and 0.3L/min oxygen in an induction chamber. The abdomen was shaved, the animals were placed in a supine position and the skin was disinfected with iodine solution. After transverse laparotomy the distal part of the common extrahepatic bile duct below the bile ducts of the inferior liver lobes (caudate inferior lobe and right inferior lobe) was identified and separated from surrounding fat tissue. Special care was taken to avoid any injury of the pancreas tissue. Three ligatures were placed around the prepared segment of the common bile duct. The common bile duct was always transected between the middle and distal ligature. Closure of the abdominal wound was always done by two-layer running suture (Prolene 6-0, Ethicon) [18].

*Postoperative care and analgesic treatment of the animals:*

Analgesic treatment was started immediately after wound closure in all animals. Buprenorphine (0.005mg/kg BW, Temgesic®) was subcutaneously injected; the analgetic therapy was given twice a day during the first three postoperative days. During this time the animals were checked for their clinical condition twice per day; afterwards the animals were routinely checked once per day. Clinical scoring was performed according to Hawkins [22].

**Histology and Immunohistochemistry:**

***Haematoxylin-eosin staining (HE)***

The samples were fixed in 4.5% buffered formalin for 48h. Sections of 4μm thickness were cut after paraffin embedding. Slides were stained with Haematoxylin-Eosin (HE) for histo-pathological examination. After staining, all slides were digitalized using a slide scanner (Nanozoomer 2.0 HT scanner and the software NDP.scan 2.3; Hamamatsu City, Japan).

Number and relative area of periportal fields as well as the ductular reaction (diameter and number of bile ducts in periportal area) were evaluated with the measuring tool of NPG-Viewer (“NanoZoomer Digital Pathology”; Hamamatsu, Japan). Results were given as numerical value, for size in mm², and relative size in %. The relative area represents the area of ductular reaction in relation to area of the total section [%].

***Bromodeoxyuridine staining (BrdU)***

The staining procedure based on a modified protocol of Sigma Inc. After deparaffinization and rehydration, tissue sections were treated with prewarmed 0.1% trypsin solution at 37°C for 20 minutes, followed by denaturation with 2 N HCl at 37°C for 30 minutes, and blocking with avidin solution for 10 minutes, biotin solution for 10 minutes, and 5% goat serum BSA-TBS at 37°C for 15 minutes. In the next step sections were incubated with 1:50 monoclonal anti-BrdU antibody (DAKO Inc.) at 37°C for 1 hour, followed by 1:300 biotinylated Fab-specific goat anti-mouse linked antibody (Sigma Inc.) for 30 minutes and AP-conjugated streptavidin (DAKO Inc.) for 30 minutes, prior to the application of Neofuchsin solution for 20 minutes. The sections were washed, counterstained with Hematoxylin, and coverslipped with Immu-Mount (Shandon Inc.).

**Results:**

**Figure 2: 3D-recos of samples with incomplete filling of the murine biliary tree after tBDT.**

**Figure 5**: **“Internal quality control” using HE-staining of MV-Samples**

**Figure 8 A-F: Results of morphometric analysis of 2D-histology** using HE and BrdU. After tBDT the relative area of bile ducts per PF increased (**A**), due to slowly increasing diameter (**B**) and primarily to strongly increasing number (**C**)of bile per portal field. The cholangiocytes showed a proliferative activity (**D**) with two peaks at day 3 and day 7 after tBDT. The hepatocellular compartment was stepwise reduced (**E**) due to the biliary enlargement following biliary occlusion. The hepatocytes showed a constantly increasing proliferative activity (**F**) until day 28 (~end of observation) after tBDT.

References

1. Hawkins P. Recognizing and assessing pain, suffering and distress in laboratory animals Laboratory Animals 2002 36, 378–395
