# Supplementary material for: Corrosion Cast and 3D Reconstruction of the Murine Biliary Tree After Biliary Obstruction: Quantitative Assessment and Comparison With 2D Histology
Source: J Clin Exp Hepatol. 2021 Dec 20;12(3):755–66. doi: 10.1016/j.jceh.2021.12.008 (PMC9168744; doi:10.1016/j.jceh.2021.12.008)

**Figure 2: 3D-recos of samples with incomplete filling of the murine biliary tree after tBDT.** In these three samples we were challenged by an enormous filling pressure although the biliary tree was decompressed as performed in the other samples. (Please note that we used abbreviations of murine liver lobes acc. to [10]: RML=right median lobe; LML=left median lobe; LLL=left lateral lobe.)


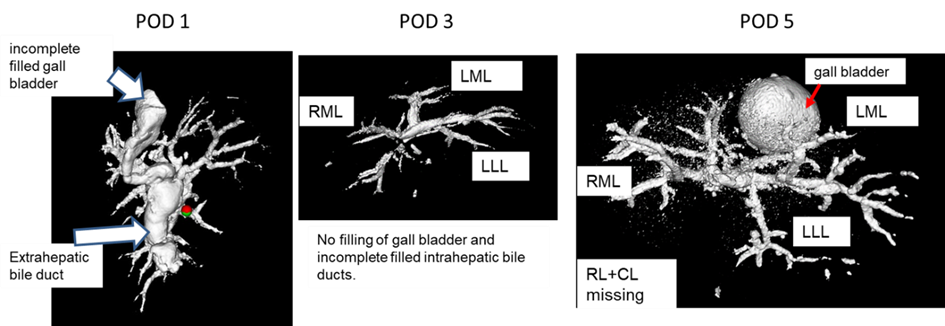

Supplement: Multimedia component 2 [file mmc2.docx]
