# Supplementary material for: Corrosion Cast and 3D Reconstruction of the Murine Biliary Tree After Biliary Obstruction: Quantitative Assessment and Comparison With 2D Histology
Source: J Clin Exp Hepatol. 2021 Dec 20;12(3):755–66. doi: 10.1016/j.jceh.2021.12.008 (PMC9168744; doi:10.1016/j.jceh.2021.12.008)

**Figure 5**: **“Internal quality control” using HE-staining of MV-Samples** to identify MV inside bile ducts and to identify extravasates. **A:** 3D-reco of the biliary tree at six time points after occlusive tBDT. The biliary tree was filled with radiopaque Microfil© (MV) for 3D-reconstruction using µCT-Scans. **B:** We identified the presence of MV in large and small intrahepatic bile ducts (black arrows) in all samples by HE-staining. One representative picture of RML (right median lobe) of the MV samples is always shown for every time point, respectively. We detected in one sample extravasates (asterisk) of MV (1xPOD3) in the liver parenchyma suggesting a ruptured terminal bile duct following injection of MV. In one sample of POD 14 we found MV in biliary convolutes and few extravasates. In the sample of POD 5 we found no MV (white arrows) in the bile ducts of RML, this correlates with the 3D-reco of RML, since we could not reconstruct bile ducts of the cranial liver lobes (RML+LML+LLL) in this sample.


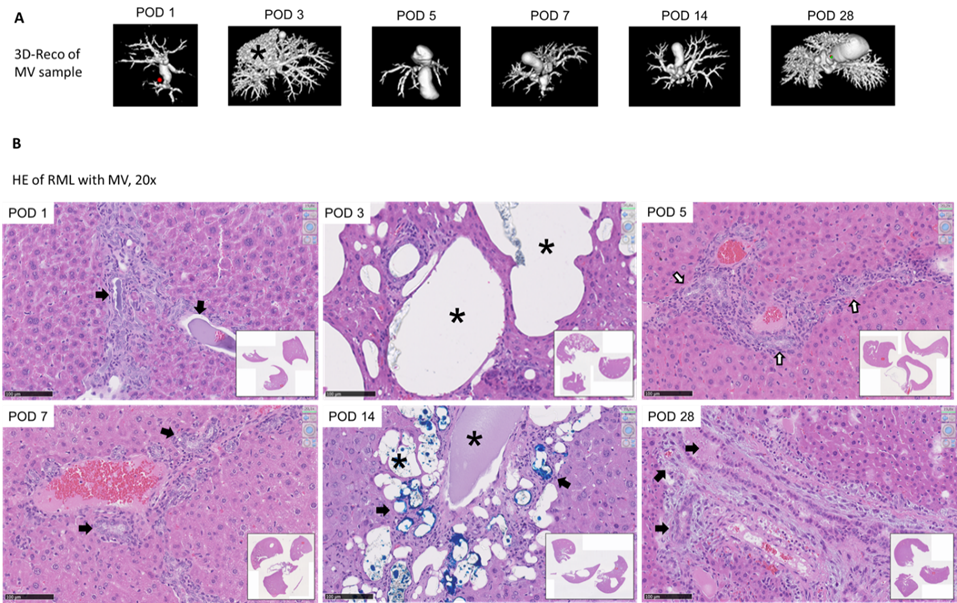

Supplement: Multimedia component 3 [file mmc3.docx]
