# Supplementary material for: Corrosion Cast and 3D Reconstruction of the Murine Biliary Tree After Biliary Obstruction: Quantitative Assessment and Comparison With 2D Histology
Source: J Clin Exp Hepatol. 2021 Dec 20;12(3):755–66. doi: 10.1016/j.jceh.2021.12.008 (PMC9168744; doi:10.1016/j.jceh.2021.12.008)

**Figure 8 A-F: Results of morphometric analysis of 2D-histology** using HE and BrdU. After tBDT the relative area of bile ducts per PF increased (**A**), due to slowly increasing diameter (**B**) and primarily to strongly increasing number (**C**)of bile per portal field. The cholangiocytes showed a proliferative activity (**D**) with two peaks at day 3 and day 7 after tBDT. The hepatocellular compartment was stepwise reduced (**E**) due to the biliary enlargement following biliary occlusion. The hepatocytes showed a constantly increasing proliferative activity (**F**) until day 28 (~end of observation) after tBDT.


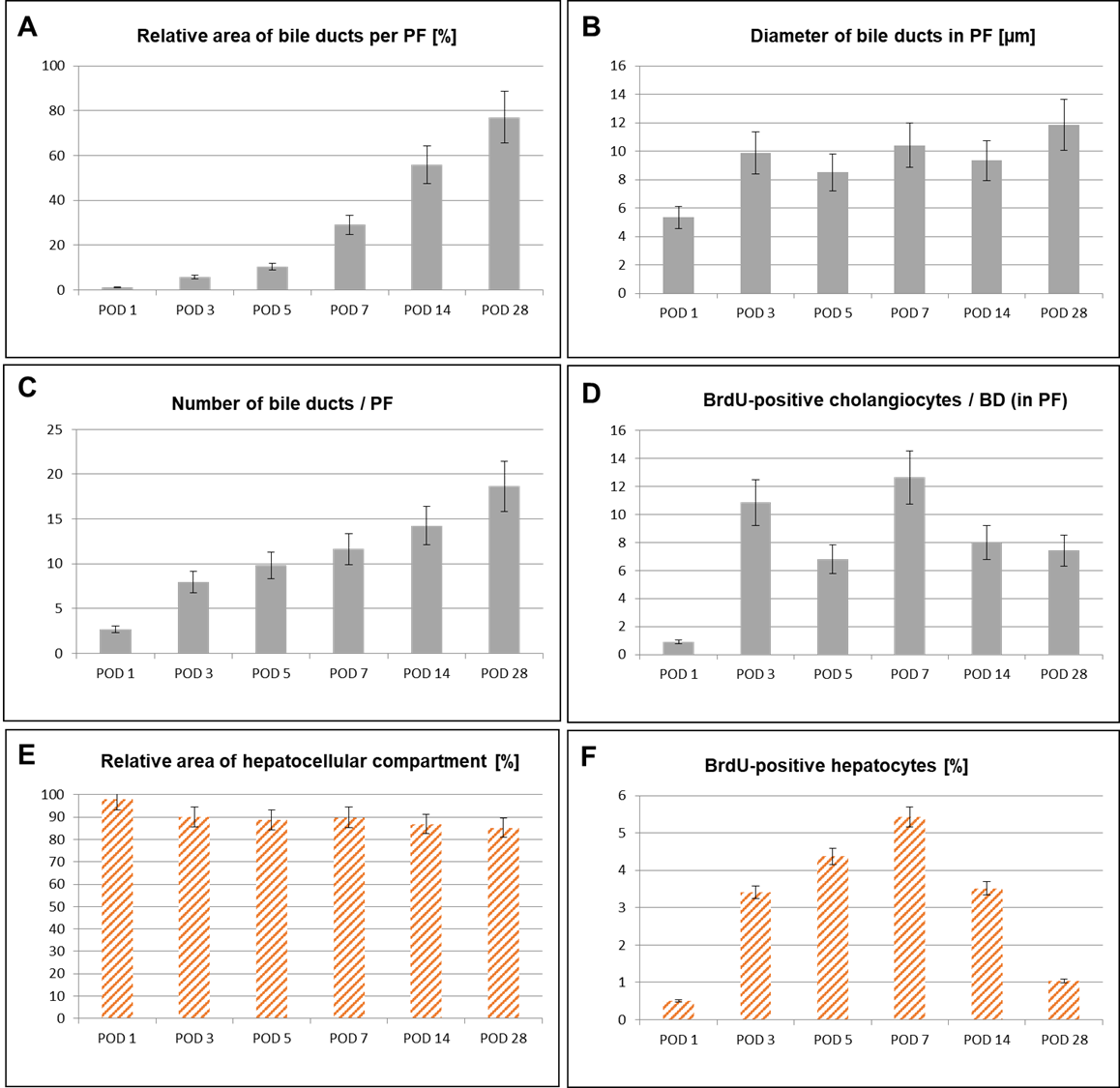

Supplement: Multimedia component 4 [file mmc4.docx]
