# Supplementary material for: Corrosion Cast and 3D Reconstruction of the Murine Biliary Tree After Biliary Obstruction: Quantitative Assessment and Comparison With 2D Histology
Source: J Clin Exp Hepatol. 2021 Dec 20;12(3):755–66. doi: 10.1016/j.jceh.2021.12.008 (PMC9168744; doi:10.1016/j.jceh.2021.12.008)
Supplement: Multimedia component 5 [file mmc5.doc]

| **Table 1:** Results of Morphology (HE) and Immunohistochemistry (BrdU) of mouse liver after tBDT (n=2 per time point). | | | | | | | | | | | | |  | |  | |  |  | |  | |  |
| --- | --- | --- | --- | --- | --- | --- | --- | --- | --- | --- | --- | --- | --- | --- | --- | --- | --- | --- | --- | --- | --- | --- |
|  | **POD 1** | | | **POD 3** | | | **POD 5** | | | **POD 7** | | | **POD 14** | | | | | **POD 28** | | | | |
|  | mean | ± | stdev. | mean | ± | stdev. | mean | ± | stdev. | mean | ± | stdev. | mean | ± | | stdev. | | mean | ± | | stdev. | |
|  |  |  |  |  |  |  |  |  |  |  |  |  |  |  | |  | |  |  | |  | |
| **Portal fields (PF)** |  |  |  |  |  |  |  |  |  |  |  |  |  |  | |  | |  |  | |  | |
| relative area of PF [%] | 1.02 | ± | 0.57 | 3.92 | ± | 1.27 | 4.78 | ± | 2.48 | 6.34 | ± | 2.01 | 7.12 | ± | | 3.25 | | 11.56 | ± | | 6.14 | |
| Diameter of bile ducts per PF [µm] | 14.36 | ± | 2.30 | 39.53 | ± | 1.63 | 31.94 | ± | 1.12 | 55.28 | ± | 1.87 | 51.57 | ± | | 1.56 | | 131.69 | ± | | 39.67 | |
| Number of bile ducts per PF | 7.75 | ± | 2.01 | 7.14 | ± | 1.75 | 8.83 | ± | 1.34 | 15.50 | ± | 3.99 | 18.33 | ± | | 8.33 | | 18.67 | ± | | 6.87 | |
| BrdU-positive BD cells per PF [%] | 0.08 | ± | 0.29 | 10.86 | ± | 3.68 | 6.83 | ± | 2.17 | 12.67 | ± | 5.19 | 8.00 | ± | | 6.44 | | 7.42 | ± | | 4.56 | |
| Relative area of bile ducts per PF [%] | 0.01 | ± | 0.57 | 5.70 | ± | 0.67 | 10.30 | ± | 4.78 | 29.00 | ± | 3.75 | 56.00 | ± | | 9.34 | | 77.00 | ± | | 9.89 | |
|  |  |  |  |  |  |  |  |  |  |  |  |  |  |  | |  | |  |  | |  | |
| **Extraportal ductular reaction** |  |  |  |  |  |  |  |  |  |  |  |  |  |  | |  | |  |  | |  | |
| relative area [%] | 0.01 | ± | 0.34 | 0.89 | ± | 0.64 | 1.79 | ± | 0.97 | 2.31 | ± | 0.93 | 2.87 | ± | | 1.12 | | 3.02 | ± | | 1.21 | |
| number of biliary convolutes | 3.00 | ± | 0.21 | 6.35 | ± | 1.82 | 10.38 | ± | 2.98 | 15.83 | ± | 3.25 | 21.45 | ± | | 5.45 | | 24.53 | ± | | 6.51 | |
| number of bd per convolute | 2.45 | ± | 0.78 | 2.76 | ± | 1.56 | 3.14 | ± | 1.16 | 4.57 | ± | 1.03 | 4.37 | ± | | 1.73 | | 5.31 | ± | | 1.83 | |
| diameter of bd per convolute [µm] | 4.87 | ± | 1.67 | 6.12 | ± | 3.81 | 6.38 | ± | 2.89 | 6.75 | ± | 2.73 | 8.98 | ± | | 2.91 | | 9.01 | ± | | 3.01 | |
| BrdU-positive BD cells per convolute [%] | 4.36 | ± | 0.56 | 15.52 | ± | 2.71 | 12.94 | ± | 2.71 | 9.82 | ± | 1.83 | 11.98 | ± | | 2.58 | | 6.03 | ± | | 1.36 | |
|  |  |  |  |  |  |  |  |  |  |  |  |  |  |  | |  | |  |  | |  | |
| **Hepatocytes** |  |  |  |  |  |  |  |  |  |  |  |  |  |  | |  | |  |  | |  | |
| relative area [%] | 97.99 | ± | 1.72 | 89.99 | ± | 5.24 | 89.57 | ± | 5.38 | 89.85 | ± | 3.76 | 86.88 | ± | | 4.35 | | 85.21 | ± | | 5.01 | |
| BrdU-Index of hepatocytes [%] | 0.50 | ± | 0.32 | 3.41 | ± | 0.82 | 4.38 | ± | 0.86 | 5.43 | ± | 0.65 | 3.52 | ± | | 1.01 | | 1.03 | ± | | 0.74 | |
|  |  |  |  |  |  |  |  |  |  |  |  |  |  |  | |  | |  |  | |  | |
| **Necrosis** |  |  |  |  |  |  |  |  |  |  |  |  |  |  | |  | |  |  | |  | |
| number | 10.67 | ± | 3.89 | 17.71 | ± | 5.27 | 13.96 | ± | 4.31 | 7.12 | ± | 2.32 | 17.69 | ± | | 7.35 | | 5.35 | ± | | 1.28 | |
| relative area [%] | 0.98 | ± | 0.52 | 5.2 | ± | 1.84 | 3.86 | ± | 1.60 | 1.5 | ± | 0.49 | 3.13 | ± | | 1.45 | | 0.21 | ± | | 0.34 | |
